# Cytotoxic Activities, SAR and Anti-Invasion Effects of Butylphthalide Derivatives on Human Hepatocellular Carcinoma SMMC7721 Cells

**DOI:** 10.3390/molecules201119699

**Published:** 2015-11-12

**Authors:** Yihan Hu, Xiaoxu Bi, Pu Zhao, Huachuan Zheng, Xueshi Huang

**Affiliations:** 1 Laboratory of Metabolic Disease Research and Drug Development, China Medical University, Shenyang 110001, China; huyihank@hotmail.com; 2 Institute of Microbial Pharmaceuticals, College of Life and Health Sciences, Northeastern University, Shenyang 110819, China; xiaoxu.bi@hotmail.com (X.B.); zhaopu6687700@163.com (P.Z.); 3 Cancer Research Center, Key Laboratory of Brain and Spinal Cord Injury of Liaoning Province, and Laboratory Animal Center, the First Affiliated Hospital of Liaoning Medical University, Jinzhou 121001, China; zheng_huachuan@hotmail.com

**Keywords:** *Ligusticum chuanxiong*, butylphthalides, cytotoxic activity, SAR, anti-invasion

## Abstract

A series of butylphthalide derivatives (BPDs) **1**–**8** were isolated from the extract of the dried rhizome of *Ligusticum chuanxiong* Hort. (Umbelliferae). The cytotoxic activities of BPDs **1**–**8** were evaluated using a panel of human cancer cell lines. In addition, the SAR analysis and potential anti-invasion activities were investigated. The sp^2^ carbons at C-7 and C-7a appeared to be essential for the cytotoxic activities of BPDs. BPDs **5** and **6** remarkably inhibited the migration and invasion of cancer cells. The anti-invasion activity of dimer **6** was demonstrated to be significantly higher than monomer **5**.

## 1. Introduction

Butylidenephthalide derivatives (BPDs) are characterized by a butyl at C-3 of the phthalide frameworkin monomer or dimer forms, and commonly produced in Umbelliferae plants, such as *Ligusticum chuanxiong* Hort*.*, *Angelica sinensis* (Oliv.) Diels, *Apium graveolens* L., *etc.* [1,2,3,4]. BPDs exhibit a wide variety of biological activities, including anti-inflammatory, blood flow increasing, smooth muscle relaxation and anti-tumor activities [5,6,7,8,9]. The anti-tumor potential of BPDs has been intensively studied because of its inhibition effect on the growth of human glioblastoma [10]. Although antitumor activities of some phthalide monomers have been previously explored, the systematical structure-activity relationship (SAR) of BPDs has not been investigated.

*L. chuanxiong* is a traditional Chinese herb which belongs to the Umbelliferae family, which is commonly used to promote blood circulation and to relieve rheumatism. There are a large number of biologically active substances in *L. chuanxiong*, including many kinds of butylidenephthalides [11]. In order to investigate BPDs’ SAR for cytotoxity, we isolated five BPD monomers **1**–**5** and three BPD dimers **6**–**8** from the rhizome extract of *L. chuanxiong*. The cytotoxic activities against human large-cell lung cancer cell line H460, human liver cancer cell line SMMC7721, and human gastric cancer cell line BGC823 were determined in this paper. The migration and invasion effects of **5** and **6** were evaluated on SMMC7721 cells.

## 2. Results and Discussion

### 2.1. Isolation and Structure Identification of Compounds ***1***–***8***

**Figure 1:**
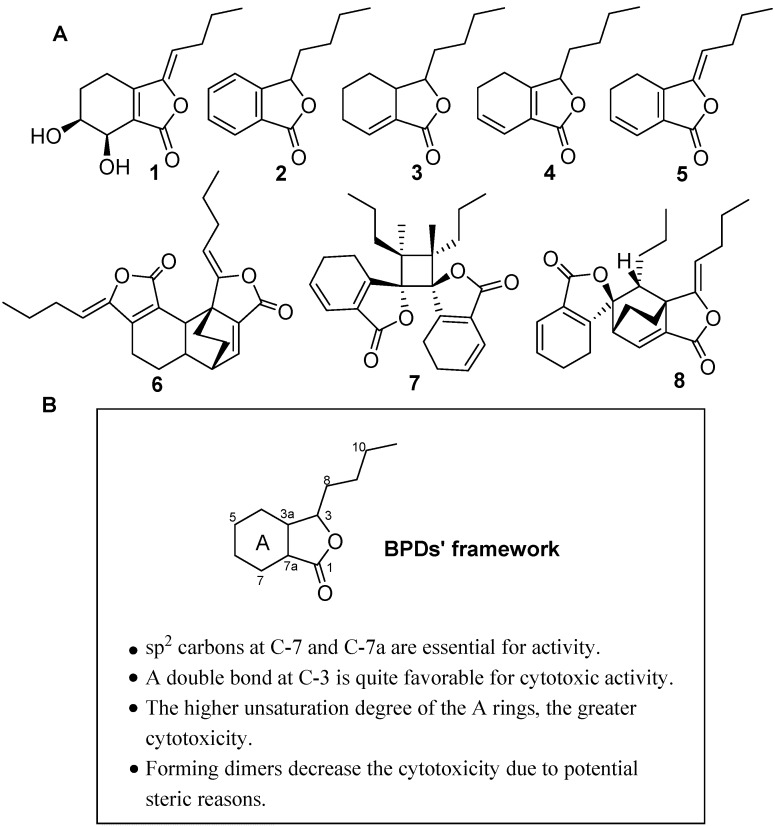
SAR analysis of the BPDs**1**–**8** from *L. chuanxiong*. (**A**) The structures of BPDs **1**–**8**; (**B**) SAR of BPDs **1**–**8**.

The dry rhizomes of *L. chuanxiong* were extracted thrice with 95% EtOH at reflux temperature. The combined crude extracts were concentrated *in vacuo* at 45 °C to remove ethanol and then partitioned between EtOAc and H_2_O. The EtOAc fraction was purified by various column chromatographies (silica gel, ODS, Sephadex LH-20) methods using different solvent combinations to yield five BPD monomers **1**–**5** and three BPD dimers **6**–**8**. Compounds **1**–**8** were identified by a combination of spectroscopic analysis and comparison with previously reported data as senkyunolide H (**1**) [12], 3-butylphthalide (**2**) [13], neocnidilide (**3**) [14], senkyunolide A (**4**) [15], (*Z*)-ligustilide (**5**) [16], 6,6′,7,3′a-diligustilide (**6**) [17], 3,3′,8,8′-diligustilide (**7**) [18], and 3a,8′,6,3′-diligustilide (**8**) [18], respectively (Figure 1A).

### 2.2. Cytotoxic Effect of BPDs on H460, SMMC7721 and BGC823 Cells

In this study, compounds **1**–**8** were subjected to cytotoxicity assays by the MTT method using three human tumor cell lines (H460, SMMC7721, and BGC823) after exposure to the different compounds for 72 h. As shown in Table 1, BPDs **2**–**8** showed different cytotoxic activities, which were influenced by the unsaturated bond in A-ring and C-3 in the BPDs’ framework (Figure 1B). These findings indicated that sp^2^ carbons at C-7 and C-7aappeared to be essential for the cytotoxcities (**2**–**8** *vs.* **1**). A double bond at C-3 is quite favorable for the cytotoxic activity (**5** *vs.* **4**). Meanwhile, the cytotoxic activities were shown to be significantly enhanced when the unsaturation of the A ring of BPDs increased (**2** *vs.* **3**, **4**). BPDs dimers were less cytotoxic compared to the monomers (**6**–**8** *vs.* **5**).

**Table 1:** The BPDs’ cytotoxicities against H460, SMMC7721, and BGC823 cell lines (IC_50_ mean ± SD, μg/mL) (*n* = 3).

Compound	H460	SMMC7721	BGC823
**1 ^a^**	-	-	-
**2**	32.0 ± 2.1	22.4 ± 1.0	25.5 ± 1.0
**3**	69.0 ± 5.3	53.3 ± 1.3	49.9 ± 2.5
**4**	86.1 ± 10.7	71.2 ± 10.1	80.7 ± 10.9
**5**	13.3 ± 0.7	3.2 ± 0.3	4.0 ± 0.1
**6**	26.9 ± 1.6	18.8 ± 1.2	55.1 ± 3.5
**7**	34.8 ± 0.5	72.5 ± 8.7	69.7 ± 10.3
**8**	6.4 ± 0.4	13.4 ± 1.0	19.1 ± 2.4

^a^ no cytotoxic activity at 100 μg/mL.

### 2.3. Effects of BPDs ***5*** and ***6*** on the Viability of SMMC7721 Cells

**Figure 2:**
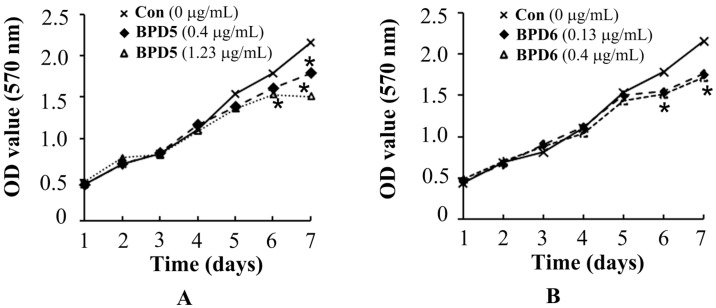
The proliferating rates of SMMC7721 cells in response to BPDs **5** and **6** treatment. Different concentration of **5** (0.4 μg/mL or 1.23 μg/mL) and **6** (0.13 μg/mL or 0.4 μg/mL) were used to treat cells (**A**,**B**). At indicated time intervals, the proliferating rate of SMMC7721 cells were determined by MTT assay (*n* = 3). *, *p* <0.05 compared to non-treated controls.

Before the experiments to explore the effects of BPDs **5** and **6** on cell adhesion, migration and invasion, we performed MTT assay to determine the appropriate dosages of **5** and **6** to avoid interference from cytotoxic effects. The SMMC7721 cells were treated with **5** and **6** at various concentrations (0, 0.4, 1.23 μg/mL for **5** and 0, 0.13, 0.4 μg/mL for **6**) for one week. Compared with the control, the viability of SMMC7721 cells was not significantly influenced at the dosage of 1.23 μg/mL of **5** and 0.4 μg/mL of **6** in three days. Only weak growth inhibition was observed after 4 days at the maximum dosages for compounds **5** and **6** (Figure 2). The proliferating rates confirmed that the influence of **5** and **6** on cell proliferations were negligible at the test concentration.

### 2.4. Effects of BPDs ***5*** and ***6*** on Adhesion, Migration and Invasion in SMMC7721 Cells

**Figure 3:**
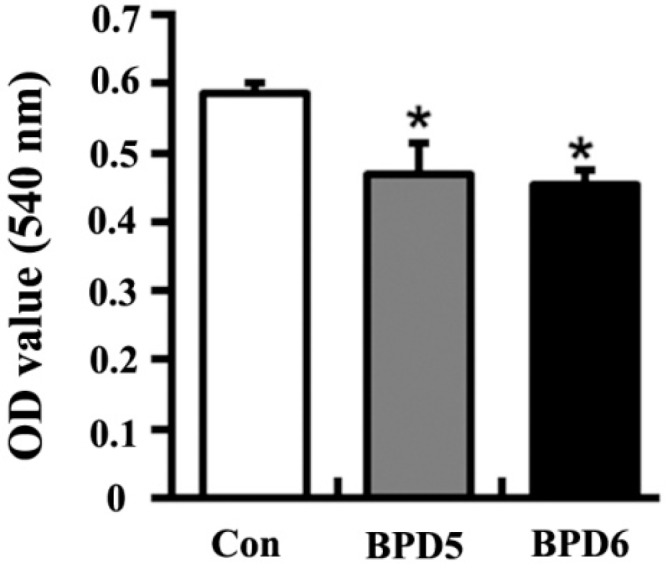
BPDs **5** or **6** inhibit the adhesion of SMMC7721 cells. The cells were seeded on matrigel with 0.5% serum-free RPMI-1640 medium before incubating with BPDs **5** (1.23 μg/mL) or **6** (0.4 μg/mL) for 1 h. The cells were washed with 2% bovine serum albumin for 30 min. The remaining cells were stained with crystal violet. The absorbance of OD at 540 nm was detected and recorded with microplate reader. Data represent as mean ± SD (*n* = 3). *, *p* < 0.05 compared with control group.

**Figure 4:**
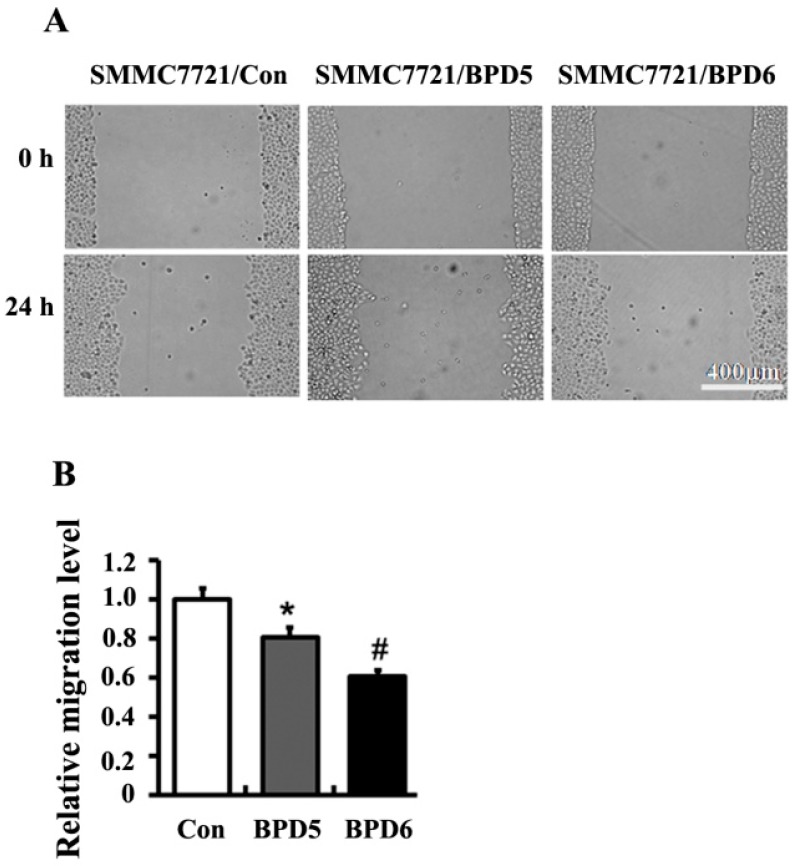
BPDs **5** and **6** suppressed the migration of SMMC7721 cells. 8 × 10^5^ cells were seeded in 6-well plates. After 24 h, the confluent cells were scratched with tips. SMMC7721 cells were treated with **5** (1.23 μg/mL) and **6** (0.4 μg/mL) for 24 h. The migration of SMMC7721 cells were determined by wound healing assay. (**A**) The images were obtained by microscopy (×20); (**B**) Relative migration levels from (**A**). Data represent as mean ± SD (*n* = 3). *, *p* < 0.05 compared with control group. #, *p* < 0.05 compared with **5** treatment.

**Figure 5:**
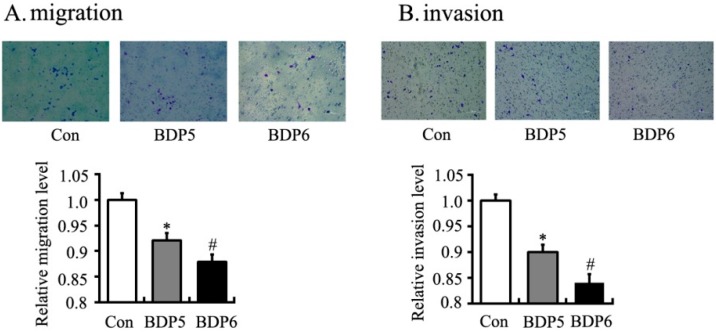
BPDs **5** and **6** decreased the migration and invasion of SMMC7721 cells. The cells were seeded in transwell upper chamber with (**B**) or without matrigel (**A**). After 24 h, the number of cells in the other side of membrane was determined by crystal staining and represented the mean ± SD (*n* = 3). *, *p* < 0.05 compared with control group. #, *p* < 0.01 compared with **5** treatment.

The effects of BPDs **5** and **6** on cell adhesion were examined by the crystal violet staining assay on SMMC7721 cells. Both BPDs **5** and **6** showed obvious anti-adhesion effects on SMMC7721 cells at 1 h (Figure 3). The migration capacities of SMMC7721 cells were significantly reduced by BPDs **5** and **6** in wound healing and transwell migration assays. In wound healing assay, **5** and **6** suppressed cell migration into the wound space (Figure 4). To further confirm the above observations, transwell experiments were carried out. The results demonstrated that the number of migrated cells was significantly reduced by treatment of **5** and **6** for 24 h (Figure 5A). In the mobility assay using transwell, similar results were obtained. **5** and **6** treatments decreased the invasive ability of SMMC7721 cells when a layer of matrigel was addedto the upper chamber of transwell (Figure 5B). In summary, the BPDs significantly inhibited the migration and invasion of SMMC7721 cells. Dimer **6** showed stronger activities than **5** in these tests (*p* < 0.05).

**Figure 6:**
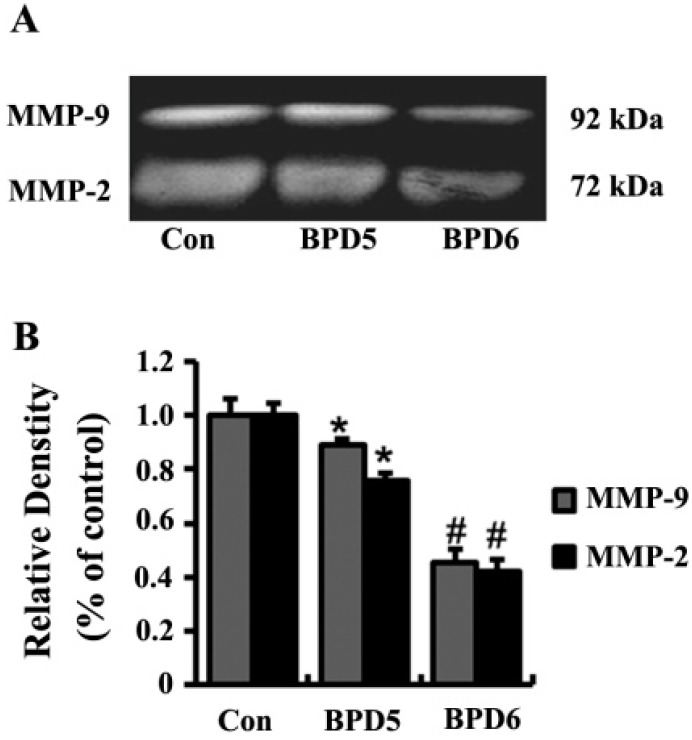
BPDs **5** and **6** inhibited MMP-2 and MMP-9 activity. MMP-2 and MMP-9 activities were determined by gelatin zymography. (**A**) The 72-kDa and 92-kDa correspond to MMP-2 and MMP-9, respectively; (**B**) MMP-2 and MMP-9 activities were quantified by densitometric analysis. Results are the mean ± SD from three independent preparations. *, *p* < 0.05 compared with control group. #, *p* < 0.01 compared with **5** treatment.

Matrix metalloproteinase-2 (MMP-2) and matrix metalloproteinase-9 (MMP-9) degrade extracellular matrix proteins and play important roles in the migration and invasion of cancer cells. In order to explore the primary anti-invasive mechanisms of BPDs, we examined the activities of MMP-2 and MMP-9 in culture media of SMMC7721 cells by zymographic assay. Compared to the control group, MMP-2 and MMP-9 activities were significantly reduced by **5** and **6** treatments (Figure 6).

## 3. Experimental Section

### 3.1. General Experimental

The NMR spectra were recorded on Bruker ARX-300 or Bruker AV-600 spectrometers. ESIMS were recorded by a LC/Triple Quadrupole Mass Spectrometer equipped with an Agilent 1290 analytical HPLC and 6420 triple quadrupole MS. Column chromatography (CC): Silica gel (100–200, 200–300 and 300–400 mesh, Qingdao Marine Chemical Ltd., Qingdao, China), Sephadex LH-20 (GE Healthcare Biosciences AB, Uppsala, Sweden), and YMC*GEL ODS-A (S-50 μm, 12 nm, YMC Co., Ltd., Kyoto, Japan) were used for column chromatography. Unless otherwise specified, all chemicals and solvents were purchased from Sinopharm Chemical Reagent Co., Ltd. (Shenyang, China) silica gel (100–200 mesh and 200–300 mesh; Qingdao Marine Chemical Ltd.), Sephadex LH-20 (GE Healthcare Biosciences AB), and YMC*GEL, ODS-A (S-50 mm, 12 nm; YMC Co., Ltd.). The MTT assay were recorded on a BioTek spectrophotometer (BioTek Instruments, Inc., Winooski, VT, USA). RPMI 1640 medium (RPMI1640), fetal bovine serum (FBS),penicillin-streptomycin-amphotericin (PSA) and 0.25% trypsin(EDTA) were purchased from Gibco BRL Co., Ltd. (Gaithersburg, MD, USA). Matrigel matrix basement membrane and albumin bovine V (BSA) were purchased from BD Biosciences (Becton, Dickinson and Company, San Diego, CA, USA). Transwell chamber were purchased from Thermo Fisher Scientific (Waltham, MA, USA). The H460, SMMC7721, BGC823 cell lines was provided by the cell bank of type culture collection of the Chinese Academy of Sciences (Shanghai, China).

### 3.2. Isolation and Preparation of Compounds

The dried rhizome of *L. chuanxiong* was purchased from Hebei Zhenyu Anguo Pharmaceutical Co., Ltd. (Anguo, China) and a voucher specimen (MPI-003#) was deposited at our laboratory. The powdered *L. chuanxiong* (10 kg) extracted three times with 95% aqueous ethanol (4 times of the materials) by reflux (each extraction period 2 h). The solvent was evaporated under vacuum to afford the crude extract*.* The extract was then suspended in water and partitioned withethyl acetate. The dried extract was subjected to silica gel column chromatography, eluting with a gradient of P.E.-EtOAc (100:1~1:10) to yield 12 fractions. By using a series of chromatographic techniques, such as silica gel column chromatography (200–300 mesh), Sephadex LH-20 chromatography and ODS chromatography, compound **3** (12 mg), **4** (16 mg) and **5** (600 mg) were isolated from fraction II, compound **2** (65 mg), **6** (300 mg), **7** (12 mg) and **8** (10 mg) were isolated from fraction III and compound **1** (12 mg) was purified from fraction IV.

### 3.3. Cytotoxicity Assay

The human cancer cell lines were cultured in RPMI-1640 medium supplemented with 10% heat-inactivated fetal bovine serum (FBS). The cytotoxic effects were evaluated by MTT method after exposure to the different compounds for various periods of time. The final concentrations of compounds **1**–**8** in the medium for cytotoxic assay were 100, 33, 10, 3.3 and 1 μg/mL, respectively. After incubation at 37 °C for 72 h, 10 μL of MTT reagent (5 mg/mL) was added to each well and incubated for 4 h, and then liquid in the wells was removed. DMSO (150 μL) was added to each well. The absorbance was recorded on a microplate reader at wavelength of 570 nm, and the IC_50_ was defined as 50% reduction of absorbance compared with the control assay.

### 3.4. Cell Adhesion Assay

The matrigel with 0.5% serum-free RPMI-1640 medium was seeded at 100μL/well in 96-well plates overnight. Then the plates were washed and incubated with 2% bovine serum albumin for 30 min. The SMMC7721 cells were seeded at 2 × 10^4^ cells/well in 96-well plates, and treated with compounds **5** and **6** for 1h. After that, the nutrient solution was removed and 0.5% crystal violet solution was added. After incubation for 15min, 100 μL 5% SDS was added to each well and the absorbance of OD at 540 nm was detected and recorded with a microplate reader.

### 3.5. Wound Healing Assay

SMMC7721 cells were seeded at 8 × 10^5^ cells/well in 6-well plates overnight. We evenly drew a straight line in the each well of 6-well plates and serum-free RPMI-1640 medium was added with compounds **5** and **6**, respectively. After incubation for 24 h, microscopic pictures were collected by an inverted microscope equipped with epifluorescence (Leica DMI3000B, Heidelberg, Germany) and analyzed with Image J software.

### 3.6. Transwell Chamber Assay

300 μL RPMI-1640 medium with 10% FBS were added in the down-room of each milli cell 24-well plates. 1 × 10^5^ cells treated with compounds **5** and **6** were suspended in 300 μL RPMI-1640 medium with 0.1% FBS and plated in the up-room of milli cell 24-well plates. After incubation for 24 h, the cells were stained by crystal violet. Microscopic pictures were collected and analyzed. For invasion assay, the matrigel with 0.3% serum-free RPMI-1640 medium as seeded at 80 μL/well in each up-room of milli cell 24-well plates incubated for 24 h and the other procedure was the same as migration assay.

### 3.7. Gelatin Zymography

The SMMC7721 cells, treated with 1.23 μM of **5** and 0.4 μM of **6**, were seeded at 2 × 10^5^ cells/well in 24-cell plates and incubated for 24 h. The supernatants were collected and subjected to electrophoresis by 10% SDS-PAGE containing 0.1% gelatin. The gels were washed thrice with 2.5% Triton X-100 and rinsed twice with floating lotion (50 mM Tris-HCl, 5 mM CaCl_2_ and 1 µM ZnCl_2_), then incubated in reaction buffer (50 mM Tris-HCl, 5 mM CaCl_2_, 1 µM ZnCl_2_ and 0.02% Brij-35) for 18 h at 37 °C. Then the gels were stained with coomassie brilliant blue R-250 for 1 h. The activities of MMP-2 and MMP-9 were quantified by densitometer measurement using a digital imaging analysis system. The densitometric analysis was performed using Image J software.

### 3.8. Statistical Analysis

The data was expressed as mean ± standard deviation. Student t test was performed using SPSS 10.0. *p* < 0.05 was considered as statistically significant.

## 4. Conclusions

In conclusion, the growth inhibition ability of BPDs against cancer cells had close relationship with their chemical structures. The presence of sp^2^ carbons at C-7 and C-7a were shown to be essential for the antitumor activities of BPDs. The unsaturated bond at C-3 increased the cytotoxic activities of BPDs. Higher unsaturation degrees of the A ring in BPDs were found to be accompanied with greater bioactivity. BPDs compounds could obviously inhibit the migration and invasion of cancer cells. Furthermore, the dimer displayed better capability to suppress tumor cell migration and invasion and less cytotoxicity than monomer. These results may be helpful for the design of future antitumor BPDs, and offer potential application in the discovery of antitumor drugs.
